# Mechanisms of mindfulness-based cognitive therapy in difficult-to-treat depression: moderation and mediation analyses from the RESPOND trial

**DOI:** 10.1017/S0033291726105212

**Published:** 2026-07-13

**Authors:** Thorsten Barnhofer, Barnaby D. Dunn, Clara Strauss, Florian A. Ruths, Mary Ryan, Asha Ladwa, Frances Stafford, Roberta Fichera, Isabella Metcalfe, Allan H. Young, Kimberley Goldsmith

**Affiliations:** 1 School of Psychology, University of Surrey, Guildford, UK; 2 Mood Disorders Centre, University of Exeter, Exeter, UK; 3 Sussex Partnership NHS Foundation Trust, Hove, UK; 4 School of Psychology, University of Sussex, Brighton, UK; 5 South London and Maudsley NHS Foundation Trust, London, UK; 6 Royal College of Psychiatrists, London, UK; 7 Devon Partnership NHS Trust, Exeter, UK; 8 Division of Psychiatry, Imperial College London, London, UK; 9 Institute of Psychiatry, Psychology and Neuroscience, King’s College London, London, UK

**Keywords:** decentering, depression, difficult-to-treat, mindfulness-based cognitive therapy, moderated mediation, moderation, mediation, mechanisms, symptom severity

## Abstract

**Background:**

Mindfulness-based cognitive therapy (MBCT) was developed for relapse prevention in people with remitted depression but is increasingly used for those with difficult-to-treat depression (DTD). A key question regarding this broader application is whether ongoing depressive symptoms constrain therapeutic responsiveness or disrupt MBCT’s proposed mechanism, decentering. We explored whether baseline depressive severity moderates clinical outcomes, whether changes in decentering mediate treatment effects, and whether this mediation varies by baseline severity.

**Methods:**

Secondary moderation, mediation, and moderated mediation analyses were conducted using data from the RESPOND randomized trial (*N* = 234), comparing MBCT plus treatment as usual (TAU) with TAU alone in adults not remitted after high-intensity psychological therapy. Depressive symptoms (PHQ-9) and decentering (Experiences Questionnaire) were assessed at baseline, post-treatment (10 weeks), and follow-up (34 weeks). Analyses were conducted using structural equation modelling.

**Results:**

Higher baseline severity predicted greater symptom improvement across both groups. Treatment-related increases in decentering partially mediated the effect of MBCT on depressive symptoms at follow-up. Although baseline severity did not moderate the treatment effect, it moderated the indirect effect, with decentering more strongly associated with symptom reduction among those with higher baseline depression. Severity did not moderate the acquisition of decentering skills.

**Conclusions:**

Concerns that more severe depressive symptoms limit the effectiveness of MBCT were not supported. MBCT’s core mechanism remained operative under substantial symptom burden, with clinical impact amplified at higher severity. These findings reduce key uncertainties regarding the application of MBCT in DTD and support its use across a broad range of symptom severity.

## Introduction

Depression that fails to remit following evidence-based treatment is common and represents a major public health challenge (McIntyre et al., 2023). Such presentations, increasingly conceptualized as difficult-to-treat depression (DTD), are associated with substantial functional impairment, high levels of service use, and ongoing clinical risk (McAllister-Williams et al., 2020; Rush et al., 2022). Contemporary guidance emphasizes the need for sustained, multimodal approaches that extend beyond symptom reduction alone (McAllister-Williams et al., 2020). Despite this, psychological treatments remain underutilized in this population (Markowitz et al., 2022). There is now evidence that mindfulness-based cognitive therapy (MBCT), a group intervention combining elements of cognitive therapy with training in mindfulness meditation, can be effective in populations meeting conventional definitions of DTD (Barnhofer et al., 2009, 2025; Chiesa et al., 2015; Cladder-Micus et al., 2018; Eisendrath et al., 2016; Garcia et al., 2023; Michalak, Schultze, Heidenreich, & Schramm, 2015). However, guidance on evaluating complex interventions in new contexts emphasizes moving beyond questions of effectiveness to consider for whom and how the intervention works, and how evidence can inform real-world decision making (Moore et al., 2021; Skivington et al., 2021). A key uncertainty in applying MBCT to DTD arises from the fact that MBCT was originally developed to prevent relapse in patients who had recovered from depression. Early guidance cautioned against its use during active depressive episodes due to concerns about cognitive and motivational demands. It is therefore important to determine whether current symptom severity, especially across the moderate to severe range, influences treatment effects and how it interacts with the psychological mechanisms through which MBCT exerts its clinical benefits.

According to the program theory of MBCT, the core mechanism underlying its effects is the cultivation of decentering: the capacity to observe thoughts and emotions as transient mental events rather than as accurate reflections of the self or reality (Teasdale, 1999; Teasdale, Segal, & Williams, 1995). In trials of MBCT for relapse prevention, increases in decentering have consistently been shown to mediate treatment effects on depressive outcomes (Bieling et al., 2012; Dimidjian et al., 2023; Segal et al., 2019). Current depressive symptoms may influence this learning process in different ways. On the one hand, elevated symptom severity may undermine learning from mindfulness practice and impede the acquisition of decentering skills. On the other hand, decentering is theorized to buffer negative mood and stress (Creswell & Lindsay, 2014; Lindsay & Creswell, 2017), suggesting that its beneficial effects may be more pronounced under conditions of greater symptom burden, potentially facilitating learning and clinical impact. Consistent with this latter view, evidence from relapse-prevention studies suggests that MBCT effects are stronger in individuals with higher residual symptoms (Kuyken et al., 2016), and a recent secondary analysis of a relapse prevention trial (Montero-Marin et al., 2025) found that mediation of treatment effects by mindfulness skills was more evident at higher levels of residual symptoms. However, this pattern may partly reflect limited symptom change among participants entering the trial with no or very few symptoms. To date, mediation effects have not yet been examined across the wider range of depression severity encountered in difficult-to-treat populations, nor have their mechanistic underpinnings been examined directly by testing moderating effects of symptom severity on different components of the mediation pathway.

The present paper reports secondary analyses of the recently published RESPOND trial (Barnhofer et al., 2025), designed to address these questions. RESPOND compared MBCT with treatment as usual (TAU) in depressed patients who had not remitted following a full course of high-intensity psychological therapy in NHS Talking Therapies services across England. Symptoms and decentering skills were assessed at baseline, post-treatment (10 weeks post-randomization), and follow-up (34 weeks post-randomization). The trial demonstrated significant advantages of MBCT at follow-up in reducing depressive symptomatology, with small to moderate effect sizes, alongside significantly greater increases in decentering skills in the MBCT group. The availability of three assessment points allowed moderation, mediation and moderated mediation analyses to be conducted while taking temporal precedence into account.

To investigate the influence of current symptoms on clinical outcome and the key mechanism of action of MBCT, we examined three interrelated questions. First, we tested whether baseline depressive symptom severity moderated the effect of MBCT on depressive symptoms at follow-up (i.e., moderation of the treatment effect). Second, we investigated whether treatment-related increases in decentering mediated the effect of MBCT on depressive outcomes at follow-up, as observed in relapse-prevention trials (i.e., mediation). Third, we examined whether this mediating pathway was itself moderated by baseline depressive symptom severity (i.e., moderated mediation). Importantly, this allowed us to differentiate between the moderation of the overall treatment effect and the moderation of specific components of underlying mechanisms. By testing moderation effects on different paths of the mediation model, we distinguished between the moderation of the acquisition of decentering skills (a path) and the moderation of the impact of decentering on subsequent depressive symptoms (b path), directly mapping onto the competing theoretical accounts outlined above.

## Method

This is a secondary mediation analysis of the RESPOND trial using the primary outcome of the trial and the core putative mediator of MBCT, decentering skills, measured as part of the study. RESPOND was a UK-based, parallel, randomized, controlled, superiority trial that investigated the clinical effectiveness and cost-effectiveness of MBCT after non-remission with psychological therapy for depression (Barnhofer et al., 2025). Assessments took place at baseline, post-treatment (10 weeks post-randomization) and follow-up (34 weeks post-randomization), allowing temporal precedence to be considered in mediation analyses. We report findings according to the Guideline for Reporting Mediation Analyses of Randomized Trials and Observational Studies (AGReMA; Lee et al., 2021). The trial was prospectively registered with ISRCTN (ISRCTN17755571), on 2 February 2021.

### Participants

The sample consisted of participants aged 18 or over who had not remitted after receiving at least 12 sessions of NHS Talking Therapies high-intensity evidence-based treatment, as reflected in a Patient Health Questionnaire-9 (PHQ-9; Kroenke, Spitzer, & Williams, 2001) score of at least 10 at the end of the treatment. All participants met the criteria for Major Depressive Disorder at entry into the trial according to a structured clinical interview. Potential participants were excluded, if they: were eligible for secondary care services; presented with a level of risk to self or others that could not be safely managed in a primary care service context; had a history of psychotic symptoms, current mania, alcohol or substance use disorder or dependence within the past 3 months, current post-traumatic stress disorder, obsessive-compulsive disorder, and/or eating disorder; had any other clinically significant condition that might have put them at risk, or might have affected the result of the trial or their ability to participate in the trial. The sample size of the study was determined for testing clinical effectiveness, and not for (moderated) mediation analyses, which therefore need to be seen as exploratory. The rationale for investigating decentering as the core mechanism of MBCT is outlined in the protocol (Barnhofer et al., 2023).

### Randomisation and masking

Individual participants (*N* = 234) were allocated (1:1) to either MBCT + TAU or TAU alone through remote random assignment at the Exeter Clinical Trials Unit. Minimization on depression severity (PHQ-9 score 10–18 vs ≥19), antidepressant use at baseline (yes vs no), and recruitment site (Devon vs London vs Sussex) was used to support balance across groups. As baseline assessment of participants was done before random assignment, there was no risk of disclosure of treatment allocation to the assessor at the time. Use of remote assessments ruled out any potential effects of assessors on assessments of outcomes at post-treatment and follow-up. Owing to the nature of the intervention, masking was not possible for therapists, participants, and trial administrators.

### Procedures

#### Recruitment

Three research sites (Devon, Sussex, London) recruited participants from 20 NHS Talking Therapies services located across a range of rural and urban areas. Participating services identified people nearing the end of high-intensity treatment who were still reporting depressive symptoms in the clinical range (PHQ-9 score of 10 or higher) or people who, within a 6-month window after the end of high-intensity therapy, reported depressive symptoms in the clinical range on the PHQ-9 without previous remission. Interested people were contacted for initial screening, and if positive, were invited to take part in a structured clinical interview. Eligible participants completed baseline assessments using web-based questionnaires within a window of 4 weeks before random assignment. Informed consent was taken before the start of the clinical interview by asking participants to sign and return a consent form electronically.

#### Interventions

The MBCT program was delivered in line with the standard manual (Segal, Williams, & Teasdale, 2013), with a small number of modifications specific to the trial. These were minor and addressed the fact that participants were currently depressed rather than in remission, including careful monitoring for signs of emotional contagion and a more proactive stance in structuring the session (for details see Supplementary Material S1). The intervention involved an individual orientation session followed by eight weekly group meetings. The early sessions emphasized building mindfulness skills, while the later sessions focused on applying these skills to manage challenging emotional experiences more effectively. Attendance at four or more sessions was defined as the minimum level of exposure for participants to be considered as having received an adequate dose.

Sessions were led by experienced MBCT therapists, using secure videoconferencing software (Zoom Professional). Groups were intended to include around 13 participants, with actual numbers ranging from 8 to 16. Five cohorts took part between October 2021 and March 2023. All therapists met UK Good Practice Guidelines and had substantial experience teaching MBCT (M = 11.6 years, SD = 3.9). Prior to the trial, therapists attended a one-day training to familiarize themselves with the adapted protocol. They received supervision from an experienced MBCT teacher (TB) following the orientation session and after each weekly class. Adherence to the treatment protocol was evaluated using the MBCT Adherence Scale (Segal, Teasdale, Williams, & Gemar, 2002), drawing on material from all eight sessions, while therapist competence was assessed with the Mindfulness-Based Interventions: Teaching Assessment Criteria (MBI:TAC) based on video recordings from at least two sessions per therapist (Crane et al., 2013).

Participants in both arms were asked to continue their usual care as recommended by their general practitioner. No restrictions were placed on TAU, except that those allocated to MBCT + TAU were encouraged not to seek additional psychological therapy during the intervention period. To minimize resentful demoralization, participants in the TAU alone arm were invited to a brief interview shortly after randomization that emphasized the value of their involvement. This interview, as well as the MBCT orientation meeting, took place within 2 weeks of randomization. After trial completion, participants receiving TAU alone were offered guidance on further treatment options appropriate to their diagnosis.

#### Measures

Self-report measures were collected via web-based questionnaires at baseline, post-treatment, and follow-up.

Decentering, identified a priori as the core skill through which MBCT was expected to influence depressive symptoms (Barnhofer et al., 2023), was assessed using the Decentering subscale of the Experiences Questionnaire (EQ; Fresco et al., 2007). This measure captures the ability to observe thoughts and emotions as transient mental events rather than as literal truths or accurate reflections of the self. Higher scores reflect a greater capacity for this metacognitive, non-reactive stance. Internal consistency of the scale was Cronbach’s *α* = 0.79 at baseline, 0.84 at post-treatment, and 0.87 at follow-up. In the mediation analyses, treatment-related changes in decentering were examined as the hypothesized pathway linking MBCT+TAU to subsequent improvements in depressive symptoms relative to TAU only.

Depression severity was assessed using the Patient Health Questionnaire–9 (PHQ-9) at 34 weeks post-randomization (follow-up), which constituted the primary clinical outcome for the trial. The PHQ-9 comprises nine items corresponding to DSM-5 criteria for major depression, yielding a total score from 0 to 27, with higher values indicating more severe symptoms (Kroenke, Spitzer, & Williams, 2001). Scores of 10 or greater are commonly used as a threshold for probable depression, and a reduction of approximately six points is typically regarded as a reliable clinically meaningful improvement. Internal consistency of the scale was Cronbach’s *α* = 0.66 at baseline, 0.81 at post-treatment, and 0.84 at follow-up. The PHQ-9 is the primary depression outcome used within NHS Talking Therapies services that the RESPOND trial recruited from and is supported by strong psychometric evidence, including high sensitivity and specificity. It is widely applied in clinical research, free to use, and available in multiple languages.

### Statistical analyses

All models were estimated as structural equation models using the lavaan package for R (Rosseel, 2012). Maximum likelihood with robust (Huber–White) standard errors and a scaled test statistic (MLR) was used, with full information maximum likelihood for missing data. All analyses were pre-specified two-sided with *α* = .05 and in line with the main analyses of the trial followed the intention-to-treat (ITT) approach using observed data only.

**Figure 1.**
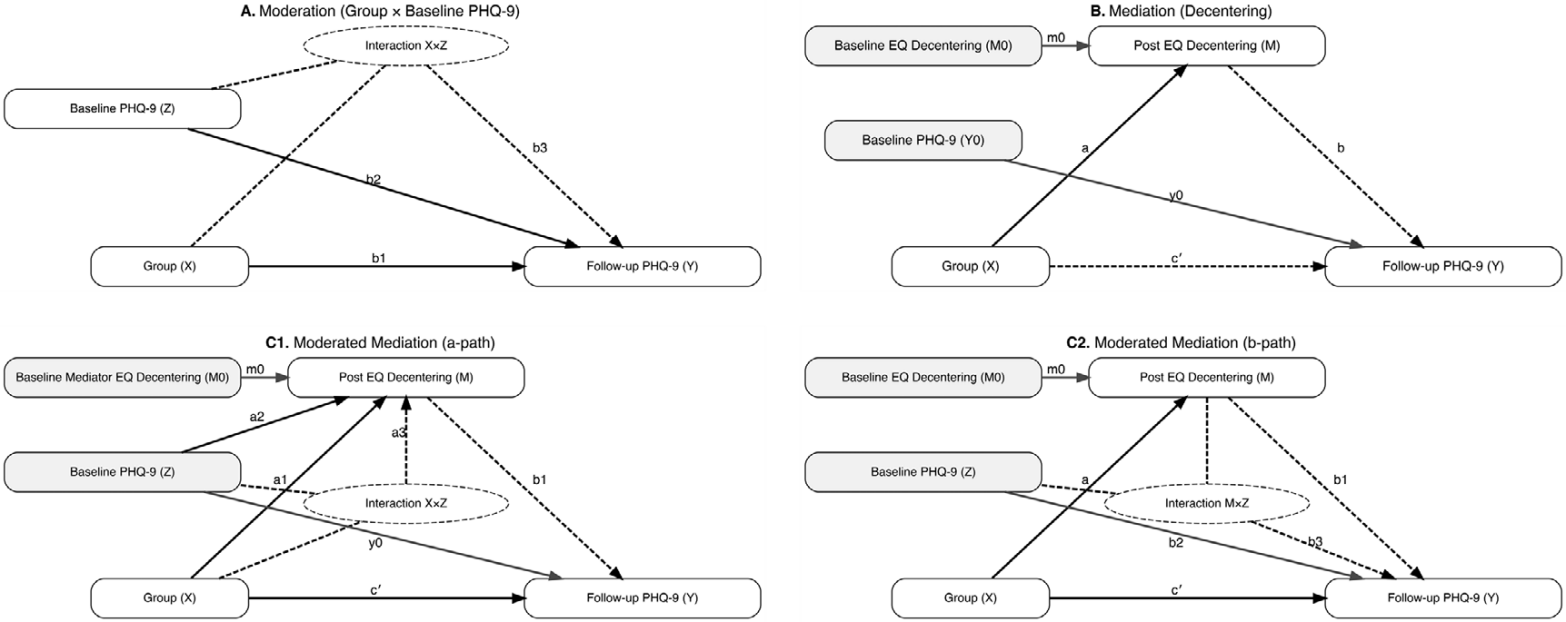
Conceptual path diagrams for moderation, mediation and moderated mediation models. *Note*: (a) The moderation model illustrates how the association between baseline depression severity and treatment outcome may differ by treatment group (Group × Baseline interaction). (b) The mediation model illustrates the indirect effect of treatment group on outcome through the putative mediator, decentering skills (paths *a* and *b*). (C1, C2) The moderated mediation model extends this framework by allowing the *a* or *b* path to vary with baseline severity, testing whether the indirect effect through decentering depends on baseline depression level. Solid lines represent structural paths that define the main regression relationships being tested. Dashed lines represent secondary, conditional or derived paths. Figure 1. long description.

The conceptual path diagrams for the moderation, mediation, and moderated mediation models are shown in Figure 1. To test whether baseline depressive symptoms moderated the effect of treatment allocation on follow-up depressive symptoms, we regressed follow-up PHQ-9 on randomized Group (0 = control, 1 = intervention), centered baseline PHQ-9, and their interaction (Group × T0 PHQ-9). Baseline PHQ-9 was mean-centered so that the Group coefficient represents the treatment effect at the average baseline severity. We probed the interaction with simple slopes of Group at the baseline mean and ± 1 SD and report the corresponding Wald tests. For visualization we plotted PHQ-9 model-implied change from T0 to T2 against baseline PHQ-9 for each Group, and marginal treatment effects at the mean and ± 1 SD.

To examine whether treatment-related improvements in decentering mediated the effect of treatment group on depressive symptoms at follow-up, we next estimated a baseline-adjusted mediation model, in which treatment group (MBCT+TAU = 1, TAU alone = 0) predicted post-treatment decentering (path *a*), which in turn predicted follow-up PHQ-9 (path *b*), controlling for baseline decentering and baseline PHQ-9 in the model for the mediator, and for the outcome, respectively (all continuous measures mean-centered; Landau, Emsley, & Dunn, 2018). Using an earlier measure of the mediator (post-treatment) and a later measure of the outcome (follow-up), is consistent with the temporal precedence hypothesis implicit in a mediation model. The direct effect of treatment on follow-up PHQ-9 (path *c′*) was estimated, with the indirect effect (*a × b*) derived, and the total effect calculated as *c′ + (a × b).* Bias-corrected bootstrap confidence intervals (2,000 resamples) were used to assess the significance of indirect effects (Fritz, Taylor, & MacKinnon, 2012). For visualization, we plotted the decomposition of total, direct (*c′*), and indirect (*a × b*) effects with 95% bootstrap confidence intervals, and a partial regression plot depicting the adjusted relation between post-treatment decentering and follow-up PHQ-9 scores (*b* path).

Finally, to assess whether the mediation effect varied according to baseline depressive severity, we tested two moderated mediation models: one in which baseline PHQ-9 moderated the *a* path (Group × baseline PHQ-9 → post-treatment decentering), and one in which it moderated the *b* path (post-treatment decentering × baseline PHQ-9 → follow-up PHQ-9). All continuous predictors were standardized prior to creating interaction terms, which were revisualized with respect to their component variables to minimize collinearity. For each model, conditional indirect effects were estimated at the baseline PHQ-9 mean and ± 1 SD, and their significance was evaluated using bias-corrected bootstrap confidence intervals (2,000 resamples). For visualization, we plotted the decomposition of total, direct (*c′*), and indirect (*a × b*) effects by baseline severity at the baseline PHQ-9 mean and ± 1 SD with 95% bootstrap confidence intervals and partial regression plots visualizing the moderated paths. Variables in the moderated mediation models were standardized to facilitate estimation of interaction terms and conditional indirect effects, while the moderation and mediation models used unstandardized variables for ease of interpretation.

Primary moderation, mediation, and moderated mediation analyses were conducted without adjustment for antidepressant use and recruitment site, as these minimization variables showed negligible effects in the main trial analyses and were therefore considered unlikely to materially influence estimates of moderation, mediation, or moderated mediation. Fully adjusted models including these covariates were estimated as sensitivity analyses and are reported in the Supplementary Material. In a further set of sensitivity analyses, both the mediator and outcome models were adjusted for baseline levels of both depressive symptoms and decentering (cross-baseline adjustment) to account for shared baseline variance and to ensure that mediation effects reflected change during treatment rather than pre-existing differences.

## Results

Of the 234 people included, 166 (70.9%) identified as women, 65 (27.7%) as men, one (<1%) as another gender, and two (0.8%) preferred not to disclose. The mean age of participants was 42.5 years (SD = 13.9). Most participants were White (201; 85.8%). Participants reported having experienced a mean of 6.08 previous depressive episodes (SD = 13.81), with onset typically in early adulthood (age of onset M = 20.30 years, SD = 10.47). The mean PHQ-9 scores at baseline were 17.95 (SD = 3.92) in the MBCT+TAU group and 17.77 (SD = 3.83) in the TAU alone group. Further details about sociodemographic and clinical characteristics of participants in the two trial groups are presented in the Supplementary Table S1. The trial recruited participants over seven cohorts. Assessments of treatment delivery indicated excellent fidelity: MBCT adherence scores averaged 31.25 (SD = 1.86) on a scale from 0 to 34, and therapist competence ratings on the MBI:TAC were at least at the competent level for all groups. Attendance across the eight MBCT group-sessions was high, with 106 of 118 participants (89.8%) attending the first session and 81 (68.6%) attending the seventh. In total, 99 participants (83.8%) received at least four sessions, representing a minimally adequate treatment dose. Follow-up outcome data were obtained for 214 participants (91.4%) at 10 weeks and 203 (86.7%) at 34 weeks; all provided corresponding EQ decentering data. Based on standard PHQ-9 severity thresholds, baseline depressive symptoms were near the upper end of the moderately severe range, with about 40% of participants meeting criteria for severe depression.

### Moderation of follow-up treatment effect by baseline PHQ-9

**Figure 2.**
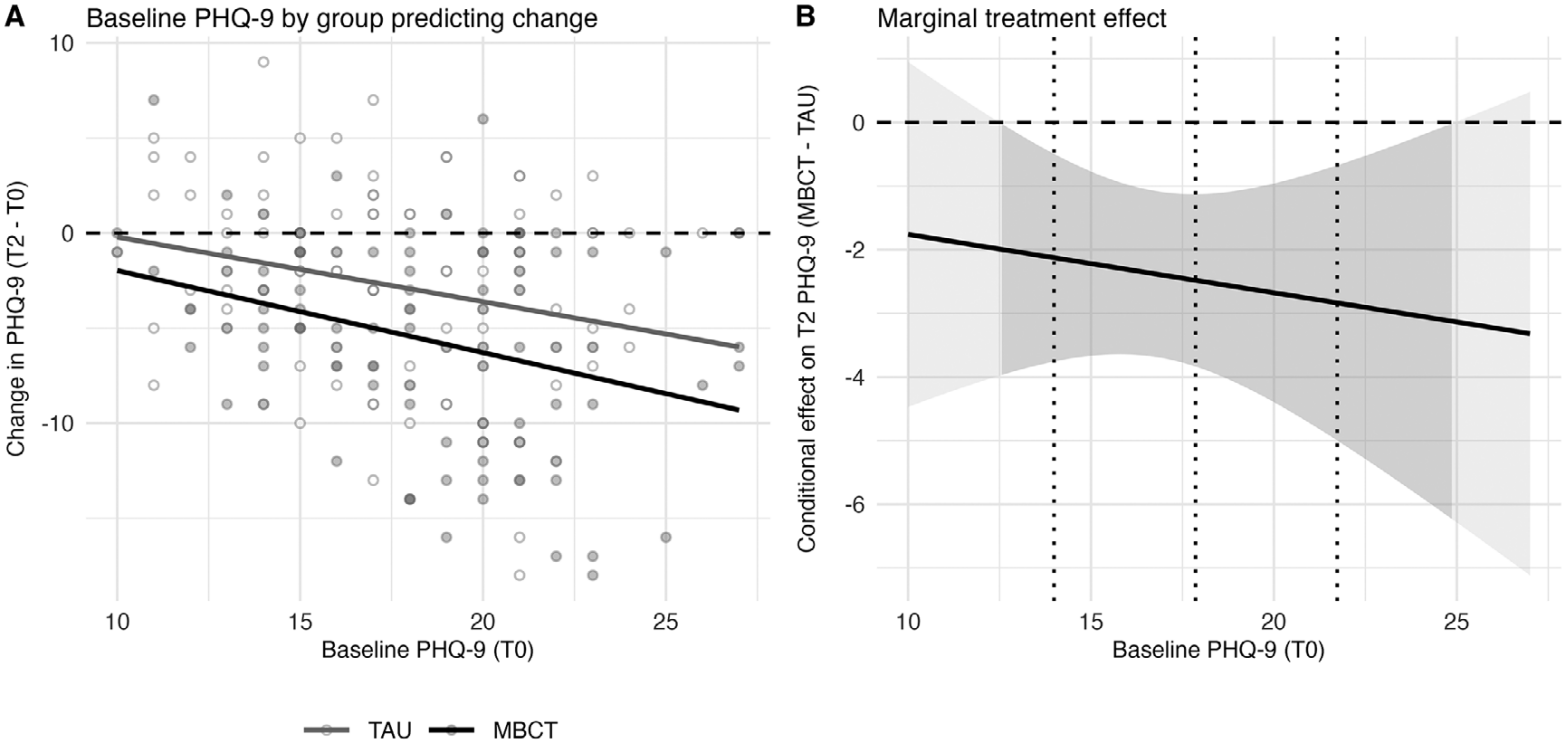
Association between baseline depression severity and treatment effects. *Note*: (a) Relationship between baseline depressive symptom severity (T0 PHQ-9) and change in depressive symptom severity (T2 – T0 PHQ-9) for participants receiving mindfulness-based cognitive therapy (MBCT; black) and treatment as usual (TAU; grey). Lines represent fitted values from an ANCOVA model; negative values indicate improvement. In both groups, higher baseline severity is associated with greater symptom improvement, with broadly similar slopes across conditions. (b) Marginal treatment effects (MBCT – TAU) on follow-up depressive symptom severity (T2 PHQ-9) across baseline severity, with 95% confidence intervals (shaded) and mean ± 1 SD (dotted). The differential treatment effect (MBCT – TAU) remains relatively consistent across the range of baseline severity. Figure 2. long description.

Results of the moderation analysis are summarized in Table 1. When controlling for baseline PHQ-9, Group had a significant effect on follow-up PHQ-9 with MBCT+TAU showing higher reductions in symptoms than TAU alone, as previously reported from the RESPOND trial.^10^ Contrary to concerns that people with more severe depression benefit less, higher baseline PHQ-9 scores were significantly associated with greater symptom improvement from baseline to follow-up in both MBCT+TAU and TAU alone (derived slope relating baseline severity and baseline-adjusted follow-up severity = *b*_2_–1 = −0.34; relations between baseline PHQ-9 scores and PHQ-9 change from baseline to follow-up are depicted in Figure 2a). Within the MBCT+TAU group, model-implied change at the average baseline was − 2.48 PHQ-9 points by follow-up, and each 1-point higher baseline was associated with ~ 0.43 additional points reduction. A similar, though numerically smaller, baseline–improvement association was seen in TAU alone (~0.34 points per baseline point). There was no evidence that this association of baseline depression severity with follow-up outcome differed by group. The Group×PHQ-9 baseline interaction was not significant and conditional treatment effects showed only small differences in magnitude at PHQ-9 baseline mean (−2.48, 95% CI −3.84 to −1.13) and at ±1 SD (−2.84, 95% CI −5.00 to −0.68; −2.13, 95% CI −3.76 to −0.50; see Figure 2b for marginal treatment effects across the entire range of baseline PHQ-9 scores).

**Table 1.** Parameter estimates, standard errors, bootstrap 95% confidence intervals and significance levels for moderation, mediation, and moderated mediation (*a*- and *b*-path) analyses Table 1. long description.

Label	Estimate	SE	95% CI	*p*
Moderation				
b1 (Group → T2 PHQ-9)	−2.48	0.69	[−3.83 to −1.12]	<.001
b2 (T0 PHQ-9 → T2 PHQ-9)	0.66	0.12	[0.41-0.90]	<.001
b3 (Group × T0 PHQ-9 → T2 PHQ-9)	−0.09	0.18	[−0.46-0.27]	.618
Mediation				
a (Group → T1 EQ Decentering)	3.45	0.61	[2.21-4.65]	<.001
m0 (T0 EQ Decentering → T1 EQ Decentering)	0.57	0.06	[0.44-0.70]	<.001
c′ (Group → T2 PHQ-9)	−1.94	0.73	[−3.37 to −0.55]	.009
b (T1 EQ Decentering → T2 PHQ-9)	−0.15	0.06	[−0.27 to −0.01]	.029
y0 (T0 PHQ-9 → T2 PHQ-9)	0.60	0.08	[0.41-0.77]	<.001
*a* × *b* (Group → T1 EQ Decentering → T2 PHQ-9)	−0.52	0.25	[−1.02 to −0.02]	.043
Moderated mediation (*a*-path)^a^				
a1 (Group → T1 EQ Decentering)	0.59	0.10	[0.38-0.81]	<.001
a2 (T0 PHQ-9 → T1 EQ Decentering)	0.06	0.05	[−0.05-0.18]	.252
a3 (Group × T0 PHQ-9 → T1 EQ Decentering)	−0.02	0.11	[−0.23-0.20]	.859
m0 (T0 EQ Decentering → T1 EQ Decentering)	0.55	0.06	[0.42-0.67]	<.001
*c*′ (Group → T2 PHQ-9)	−1.94	0.73	[−3.37 to −0.55]	.009
b1 (T1 EQ Decentering → T2 PHQ-9)	−0.86	0.39	[−1.59 to −0.05]	.029
y0 (T0 PHQ-9 → T2 PHQ-9)	2.32	0.34	[1.61-2.97]	<.001
*a* × *b* (Group → T1 EQ Decentering → T2 PHQ-9)	−0.51	0.25	[−1.02 to −0.02]	.044
Moderated mediation (*b*-path)^a^				
*a* (Group → T1 EQ Decentering)	0.60	0.10	[0.38-0.81]	<.001
m0 (T0 EQ Decentering → T1 EQ Decentering)	0.53	0.06	[0.41-0.66]	<.001
*c*′ (Group → T2 PHQ-9)	−1.93	0.72	[−3.34 to −0.59]	.008
b1 (T1 EQ Decentering → T2 PHQ-9)	−0.84	0.38	[−1.57 to −0.01]	.030
b2 (T0 PHQ-9 → T2 PHQ-9)	2.30	0.34	[1.59-2.93]	<.001
b3 (T0 PHQ-9 × T1 EQ Decentering → T2 PHQ-9)	−0.75	0.30	[−1.38 to −0.16]	.014
*a* × *b* (Group → T1 EQ Decentering → T2 PHQ-9)	−0.51	0.25	[−1.01 to −0.01]	.045

*Note*: PHQ-9, Patient Health Questionnaire 9; EQ Decentering, Experiences Questionnaire Decentering subscale.

a Coefficients in the moderated mediation models are reported on a standardized scale, which was used to facilitate estimation of interaction terms and conditional indirect effects; corresponding unstandardized estimates are reported in the primary mediation model.

Having established that treatment effects were broadly consistent across different levels of initial symptom severity, with no evidence of attenuation among participants with higher baseline symptoms, we next examined whether increases in decentering accounted for MBCT’s overall advantage in reducing depressive symptoms at follow-up.

### Mediation of follow-up treatment effect by change in decentering

**Figure 3.**
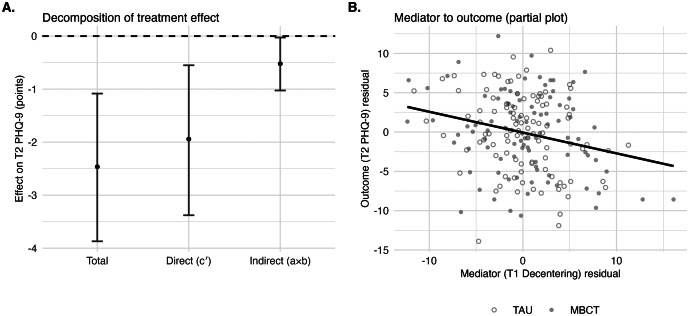
Decomposition of the treatment effect and visualization of the b path in the mediation model. *Note*: (a) Total, direct (*c*′) and indirect (*a* × *b*) effects of treatment on follow-up depressive symptom severity (T2 PHQ-9), with points indicating estimates and bars showing 95% bias-corrected bootstrap confidence intervals (2,000 resamples). The indirect effect is negative and statistically significant, indicating that increases in decentering account for part of the treatment effect on depressive symptoms. (b) Partial association between post-treatment decentering and follow-up depressive symptoms severity (T2 PHQ-9), both revisualized on baseline values and treatment group. The solid line represents the common *b* path, with open and filled symbols denoting the TAU and MBCT groups, respectively. The negative slope indicates that higher post-treatment decentering is associated with lower follow-up depressive symptoms, consistent with the mediating role of decentering. Figure 3. long description.

Results of the mediation analysis are summarized in Table 1. MBCT+TAU participants showed greater post-treatment decentering than those receiving TAU alone (*a* = 3.45, 95% CI 2.21–4.65), and higher decentering predicted lower follow-up PHQ-9 scores (*b* = −0.15, 95% CI –0.27 to −0.01). The indirect effect of treatment through post-treatment decentering was statistically significant (*a* × *b* = −0.52, 95% CI –1.02 to −0.02), indicating that part of the benefit of MBCT on depressive symptoms operated via increases in decentering (see Figure 3 for visualization). The direct effect of MBCT on follow-up PHQ-9 remained significant after accounting for decentering (*c*′ = −1.94, 95% CI –3.37 to −0.55), yielding a total effect of −2.46 (95% CI –3.87 to −1.08).

To explore whether the indirect effect of treatment via decentering varied according to baseline depressive severity, we next tested a moderated mediation model examining whether baseline PHQ-9 influenced the strength of either the *a* or *b* path.

### Moderated mediation of the a- and b-paths

**Figure 4.**
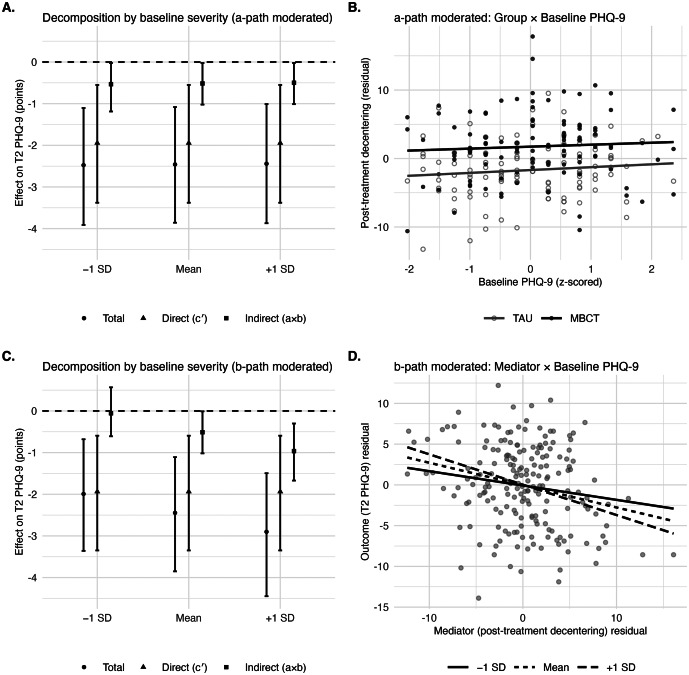
Decomposition plots and visualizations for moderated mediation models. *Note*: Panels A and C show the decomposition of the total treatment effect on depressive symptoms (T2 PHQ-9) into direct and indirect components at low (−1 SD), mean, and high (+1 SD) baseline depressive severity for models testing moderation of the *a* path (a) and the *b* path (c) by baseline PHQ-9. Points represent unstandardized effects and bars denote 95% bootstrap confidence intervals (2,000 resamples). Panel A indicates that the indirect effect via decentering is similar across levels of baseline depressive severity, consistent with no moderation of the a path. In contrast, Panel C shows that the indirect effect increases with higher baseline severity, indicating moderation of the b path. Panels B and D display partial regression plots visualizing the corresponding moderated paths: panel B shows the association between treatment group and post-treatment decentering (a path) as a function of baseline PHQ-9, and panel D shows the association between post-treatment decentering and follow-up PHQ-9 (*b* path) across levels of baseline PHQ-9. Panel B shows that the association between treatment group and post-treatment decentering (a path) does not vary as a function of baseline PHQ-9. Panel D shows that the association between post-treatment decentering and follow-up PHQ-9 (b path) becomes stronger at higher levels of baseline depressive severity, consistent with the moderated mediation effect. Figure 4. long description.

Results are presented in Table 1. In the model in which baseline PHQ-9 moderated the *a* path (Group → post-treatment decentering), the interaction between Group and baseline PHQ-9 was not significant (*a*₃ = −0.01, SE = 0.11, *p* = 0.96, 95% CI –0.22–0.22), indicating that the advantage of MBCT over TAU on post-treatment decentering did not vary with baseline depressive severity. Conditional indirect effects were similar across levels of baseline PHQ-9 (mean: –0.53, 95% CI –1.06 to −0.04; +1 SD: –0.53, 95% CI –1.08 to −0.04; −1 SD: –0.54, 95% CI –1.21 to −0.03), consistent with the absence of moderation of the *a* path (see Figures 4a and 4b).

In contrast, the model in which baseline PHQ-9 moderated the *b* path (post-treatment decentering → follow-up PHQ-9) indicated significant baseline PHQ-9 × decentering moderation (*b*₃ = −0.74, SE = 0.31, *p* = 0.01, 95% CI –1.36 to −0.15). The indirect effect of MBCT through decentering was statistically significant and strongest at higher baseline depressive severity (mean: –0.53, 95% CI –1.03 to −0.02; +1 SD: –0.98, 95% CI –1.68 to −0.29), but non-significant and smaller at lower baseline severity (−1 SD: –0.08, 95% CI –0.62–0.51; see Figure 4c,d). This pattern indicates that the mediating role of decentering was most pronounced among participants entering treatment with average to higher levels of depressive symptoms.

Results were unchanged when analyses were adjusted for minimization variables (antidepressant use and recruitment site) and when cross-baseline adjustments were included (see Supplementary Tables S2 and S3). Together, these results indicate that MBCT’s effect on depressive symptoms was partially mediated by increases in decentering, and that this indirect pathway was stronger among participants with higher baseline depression severity.

## Discussion

There is now evidence that MBCT can be effective for DTD. However, wider implementation requires evidence beyond effectiveness, including understanding of mechanisms of action and the boundaries of the treatment when used for this new indication (Moore et al., 2021; Skivington et al., 2021). The present analyses, while exploratory in nature, address a key uncertainty: how baseline depressive symptom severity is associated with clinical outcomes and how it may interact with MBCT’s core psychological mechanism.

Three main findings emerged. First, baseline symptom severity predicted clinical outcome across the trial population regardless of intervention allocation, with higher baseline symptoms associated with greater symptom improvement. Second, treatment-related increases in decentering partially mediated the effect of MBCT on depressive symptom severity at follow-up. Third, this indirect effect was moderated by baseline symptom severity, such that the association between decentering and subsequent symptom change was strongest among participants entering treatment with higher levels of depression.

Concerns that elevated symptom levels might undermine people’s capacity to benefit from MBCT have been longstanding. The present findings do not support this view. Higher baseline symptom severity was associated with greater symptom improvement in both trial groups. In the MBCT group, this association is consistent with relapse-prevention studies showing stronger effects among people with higher residual symptoms (Montero-Marin et al., 2025), and extends these observations across a spectrum that includes moderate to severe depression. These findings indicate that higher symptom burden does not attenuate, and may even be associated with stronger clinical benefits of MBCT in this context, although the present analyses do not allow firm conclusions about the extent to which increased effects reflect enhanced responsiveness to MBCT as opposed to primarily statistical factors such as greater scope for improvement and regression to the mean. Consistent with MBCT program theory, increases in decentering statistically accounted for part of the advantage of MBCT+TAU over TAU alone at follow-up. This finding is consistent with mediation results from relapse-prevention trials and suggests that the role of decentering in MBCT generalizes to the treatment of DTD. Moderated mediation analyses suggested that baseline severity did not substantially influence the acquisition of decentering skills, challenging the assumption that current depressive symptoms impede learning. In contrast, baseline severity moderated the association between post-treatment decentering and follow-up depressive symptoms, with stronger effects at higher severity levels. While the interaction estimate was associated with some uncertainty, conditional indirect effects indicated a substantially stronger mediation effect at higher baseline severity compared to lower levels. Specifically, indirect effects were close to zero at lower baseline severity but approached a one-point reduction in PHQ-9 scores at higher severity levels. This pattern is consistent with the possibility that baseline severity influences the strength with which acquired MBCT skills translate into clinical change, rather than the acquisition of those skills. These findings provide support for enhanced responsiveness under conditions of higher symptom severity. They align with the stress-buffering account of mindfulness-based interventions, which proposes that the functional role of core skills is context-sensitive and operates as a particularly potent mechanism under conditions of heightened negative affect (Creswell & Lindsay, 2014; Lindsay & Creswell, 2017). Clinically, they suggest that patients with more severe depressive symptoms should not be excluded from consideration and may, in particular, be appropriate candidates for MBCT.

### Strengths and limitations

Strengths of this study include the use of data from a large, randomized controlled trial, high treatment fidelity, and temporally ordered assessments supporting mediation analyses. Several limitations should be acknowledged. The mediation and moderated mediation analyses were secondary and exploratory, and the trial was not powered specifically to detect interaction effects. Although temporal ordering strengthens causal inference and findings were robust to sensitivity analyses, including cross-baseline adjustment, unmeasured confounding of the mediator-outcome relationship cannot be excluded. Assessments relied on self-report measures, which may be subject to shared method variance. Finally, the sample was predominantly White and recruited within UK primary care psychological therapy services, which may limit generalizability to more diverse populations or secondary care settings. Cultural factors may influence the acceptability and engagement with mindfulness-based interventions, and clinical presentations in secondary care settings are likely to be more complex, which may affect both treatment response and underlying mechanisms. The finding that decentering only partially mediated effects on depressive symptoms suggests that other specific or non-specific factors not examined in the present study contribute to the effects of MBCT, which is consistent with its conceptualization as a complex group intervention. We did not examine the extent to which changes in decentering were associated with engagement in mindfulness practice, as opposed to other aspects of the intervention, which represents an important question going beyond the scope of the current analyses. Future research should investigate the drivers of changes in decentering in more detail to better inform intervention delivery.

## Conclusion

The application of MBCT to the treatment of current DTD represents a substantive shift from its original relapse-prevention context. By demonstrating both continuity in its core psychological process and contextual modulation in its clinical impact, the present analyses support the preservation and refinement of program theory in this adapted use. Together with existing trial evidence, these results suggest that MBCT represents a viable psychological treatment option for patients with DTD and provide a clearer basis for its implementation in clinical practice.

## Supporting information

10.1017/S0033291726105212.sm001 Barnhofer et al. supplementary material Barnhofer et al. supplementary material

## Data Availability

Scientists seeking to access the data for use in future projects must do so via request to the RESPOND CI (TB) and projects using the data must have been approved in accordance with contemporary UK ethical and regulatory processes pertaining to the release of anonymized data under these circumstances. We will follow current recommendations on anonymizing and curating trial data for sharing. Trial data are stored in repositories at the site of the study sponsor (Sussex Partnership NHS Foundation Trust) and at the University of Exeter. All analytic code used in this study is available on the Open Science Framework (OSF) and will be made publicly accessible upon publication.

## Supplementary material

The supplementary material for this article can be found at http://doi.org/10.1017/S0033291726105212.

## Long descriptions

### Figure 1. Long description

The diagram consists of four panels labeled A, B, C 1, and C 2.

Panel A, Moderation. Three boxes on the left and center connect to a box on the right labeled Follow up P H Q 9 (Y). Group (X) has a solid arrow b 1 to Y. Baseline P H Q 9 (Z) has a solid arrow b 2 to Y. An oval at the top labeled Interaction X times Z has dashed arrows pointing from X and Z, and a dashed arrow b 3 pointing to Y.

Panel B, Mediation. Baseline E Q Decentering (M 0) points to Post E Q Decentering (M) via path m 0. Baseline P H Q 9 (Y 0) points to Follow up P H Q 9 (Y) via path y 0. Group (X) points to M via path a and to Y via dashed path c prime. M points to Y via dashed path b.

Panel C 1, Moderated Mediation a path. Similar to Panel B, but adds Baseline P H Q 9 (Z) and an Interaction X times Z oval. Z points to M via path a 2. The Interaction oval receives dashed lines from X and Z and points to M via dashed path a 3. Group (X) points to M via path a 1.

Panel C 2, Moderated Mediation b path. Similar to Panel B, but adds an Interaction M times Z oval. Baseline P H Q 9 (Z) points to Y via path b 2. The Interaction oval receives dashed lines from M and Z and points to Y via dashed path b 3. Group (X) points to M via path a and to Y via solid path c prime.

Navigate back to Figure 1..

### Table 1. Long description

The table is organized into five columns: Label, Estimate, S E (Standard Error), 95% C I (Confidence Interval), and p (significance level).

1. Moderation section:

- b1 (Group to T2 P H Q 9): Estimate -2.48, S E 0.69, C I [-3.83 to -1.12], p < .001.

- b2 (T0 P H Q 9 to T2 P H Q 9): Estimate 0.66, S E 0.12, C I [0.41 to 0.90], p < .001.

- b3 (Group times T0 P H Q 9 to T2 P H Q 9): Estimate -0.09, S E 0.18, C I [-0.46 to 0.27], p .618.

2. Mediation section:

- a (Group to T1 E Q Decentering): Estimate 3.45, S E 0.61, C I [2.21 to 4.65], p < .001.

- m0 (T0 E Q Decentering to T1 E Q Decentering): Estimate 0.57, S E 0.06, C I [0.44 to 0.70], p < .001.

- c prime (Group to T2 P H Q 9): Estimate -1.94, S E 0.73, C I [-3.37 to -0.55], p .009.

- b (T1 E Q Decentering to T2 P H Q 9): Estimate -0.15, S E 0.06, C I [-0.27 to -0.01], p .029.

- y0 (T0 P H Q 9 to T2 P H Q 9): Estimate 0.60, S E 0.08, C I [0.41 to 0.77], p < .001.

- a times b (Group to T1 E Q Decentering to T2 P H Q 9): Estimate -0.52, S E 0.25, C I [-1.02 to -0.02], p .043.

3. Moderated mediation a-path section:

- a1 (Group to T1 E Q Decentering): Estimate 0.59, S E 0.10, C I [0.38 to 0.81], p < .001.

- a2 (T0 P H Q 9 to T1 E Q Decentering): Estimate 0.06, S E 0.05, C I [-0.05 to 0.18], p .252.

- a3 (Group times T0 P H Q 9 to T1 E Q Decentering): Estimate -0.02, S E 0.11, C I [-0.23 to 0.20], p .859.

- m0 (T0 E Q Decentering to T1 E Q Decentering): Estimate 0.55, S E 0.06, C I [0.42 to 0.67], p < .001.

- c prime (Group to T2 P H Q 9): Estimate -1.94, S E 0.73, C I [-3.37 to -0.55], p .009.

- b1 (T1 E Q Decentering to T2 P H Q 9): Estimate -0.86, S E 0.39, C I [-1.59 to -0.05], p .029.

- y0 (T0 P H Q 9 to T2 P H Q 9): Estimate 2.32, S E 0.34, C I [1.61 to 2.97], p < .001.

- a times b (Group to T1 E Q Decentering to T2 P H Q 9): Estimate -0.51, S E 0.25, C I [-1.02 to -0.02], p .044.

4. Moderated mediation b-path section:

- a (Group to T1 E Q Decentering): Estimate 0.60, S E 0.10, C I [0.38 to 0.81], p < .001.

- m0 (T0 E Q Decentering to T1 E Q Decentering): Estimate 0.53, S E 0.06, C I [0.41 to 0.66], p < .001.

- c prime (Group to T2 P H Q 9): Estimate -1.93, S E 0.72, C I [-3.34 to -0.59], p .008.

- b1 (T1 E Q Decentering to T2 P H Q 9): Estimate -0.84, S E 0.38, C I [-1.57 to -0.01], p .030.

- b2 (T0 P H Q 9 to T2 P H Q 9): Estimate 2.30, S E 0.34, C I [1.59 to 2.93], p < .001.

- b3 (T0 P H Q 9 times T1 E Q Decentering to T2 P H Q 9): Estimate -0.75, S E 0.30, C I [-1.38 to -0.16], p .014.

- a times b (Group to T1 E Q Decentering to T2 P H Q 9): Estimate -0.51, S E 0.25, C I [-1.01 to -0.01], p .045.

Navigate back to Table 1..

### Figure 2. Long description

A two-panel figure labeled A and B.

Panel A, titled Baseline P H Q 9 by group predicting change, is a scatter plot with a horizontal x-axis labeled Baseline P H Q 9 (T 0) ranging from 10 to 25 and a vertical y-axis labeled Change in P H Q 9 (T 2 minus T 0) ranging from 10 to negative 10. A dashed horizontal line sits at 0. The plot shows two downward-sloping linear regression lines. The top line in grey represents T A U (Treatment As Usual) and the bottom line in black represents M B C T (Mindfulness-Based Cognitive Therapy). Both lines show that higher baseline scores correlate with a greater negative change (improvement) in symptoms. Individual data points are scattered around these lines as open and filled circles.

Panel B, titled Marginal treatment effect, shows the conditional effect on T 2 P H Q 9 (M B C T minus T A U) on the y-axis against Baseline P H Q 9 (T 0) on the x-axis. A solid black line slopes slightly downward from approximately negative 2 to negative 3.5 across the baseline range. This line is surrounded by a light grey shaded area representing the 95 percent confidence interval, which is narrowest in the center and widens at the extremes. Three vertical dotted lines intersect the x-axis at approximately 14, 18, and 22, representing the mean plus or minus 1 S D (Standard Deviation). A horizontal dashed line at 0 indicates the point of no differential treatment effect.

Navigate back to Figure 2..

### Figure 3. Long description

Panel A, titled Decomposition of treatment effect, is a forest plot. The y-axis is labeled Effect on T 2 P H Q-9 (points) ranging from -4 to 0. A dashed horizontal line marks the zero baseline. Three vertical error bars represent the 95 percent confidence intervals with central points for estimates. From left to right, the categories are Total, Direct (c prime), and Indirect (a times b). The Total and Direct effects have large error bars centered around -2.5 and -2 respectively, both crossing the zero line. The Indirect effect is smaller, centered near -0.5, with an error bar that does not cross zero, indicating statistical significance.

Panel B, titled Mediator to outcome (partial plot), is a scatter plot. The x-axis is labeled Mediator (T 1 Decentering) residual ranging from -10 to 10. The y-axis is labeled Outcome (T 2 P H Q-9) residual ranging from -15 to 10. The plot contains a dense cluster of open circles representing the T A U group and filled gray circles representing the M B C T group. A solid black regression line with a negative slope bisects the data, showing that as the mediator residual increases, the outcome residual decreases.

Navigate back to Figure 3..

### Figure 4. Long description

A four-panel figure labeled A through D.

Panel A. Decomposition by baseline severity (a-path moderated). The y-axis is Effect on T 2 P H Q-9 (points) ranging from -4 to 0. The x-axis shows three categories: -1 S D, Mean, and +1 S D. For each category, three points with vertical error bars are shown: a circle for Total effect, a triangle for Direct (c prime), and a square for Indirect (a times b). The indirect effect remains stable near -0.5 across all levels.

Panel B. Partial regression plot titled a-path moderated: Group times Baseline P H Q-9. The y-axis is Post-treatment decentering (residual) from -10 to 10. The x-axis is Baseline P H Q-9 (z-scored) from -2 to 2. Two nearly parallel regression lines are shown: a lower grey line for T A U and an upper black line for M B C T, indicating no interaction.

Panel C. Decomposition by baseline severity (b-path moderated). Similar axes to Panel A. The indirect effect (square) shows a downward trend, becoming more negative as baseline severity increases from -1 S D (near 0) to +1 S D (near -1).

Panel D. Partial regression plot titled b-path moderated: Mediator times Baseline P H Q-9. The y-axis is Outcome (T 2 P H Q-9) residual from -15 to 10. The x-axis is Mediator (post-treatment decentering) residual from -10 to 10. Three regression lines intersect at the origin: a solid line for -1 S D (shallowest slope), a dotted line for Mean, and a dashed line for +1 S D (steepest negative slope), showing that the relationship between decentering and outcome strengthens at higher baseline severity.

Navigate back to Figure 4..
