# Supplementary material for: Mechanisms of mindfulness-based cognitive therapy in difficult-to-treat depression: moderation and mediation analyses from the RESPOND trial
Source: Psychol Med. 2026 Jul 13;56:e228. doi: 10.1017/S0033291726105212 (PMC13370192; doi:10.1017/S0033291726105212)
Supplement: Barnhofer et al. supplementary material [file S0033291726105212sup001.docx]

**Online Supplementary Materials**

Barnhofer, T., Dunn, B. D., Strauss, C., Ruths, F. A., Ryan, M., Ladwa, A., Stafford, F., Fichera, R., Metcalfe, I. Young, A. H., & Goldsmith, K. (in submission). Mechanisms of mindfulness-based cognitive therapy in difficult-to-treat depression: moderation and mediation analyses from the RESPOND trial.

[**Supplementary Text 1. Modifications to the Original MBCT Treatment Manual** 2](#_Toc226472540)

[**Supplementary Table 1** 4](#_Toc226472541)

[**Supplementary Table 2** 6](#_Toc226472542)

[**Supplementary Table 3** 8](#_Toc226472543)

# **Supplementary Text 1. Modification to the Original MBCT Treatment Manual**

This description of modifications to the MBCT intervention is based on the protocol and supplementary materials previously reported in the RESPOND trial (Barnhofer et al., 2025), with minor adaptations for clarity.

Delivery of MBCT followed the second edition of the MBCT manual, with limited formal adaptations to account for the fact that participants were currently depressed rather than in remission. Within the overall structure of MBCT, therapists were encouraged to respond flexibly to participants’ current experiences, consistent with standard practice in enquiry and psychoeducational components. Particular attention was given to monitoring potential emotional contagion within the group, using such moments as opportunities to identify and reflect on unhelpful patterns of thinking.

Teachers were asked to embody a mindful stance throughout, while adopting a somewhat more active role in structuring sessions where needed. This included, for example, alternating between whole-group and small-group enquiries (e.g., via videoconferencing breakout rooms) and pacing discussions in a way that countered fatigue. Participants were supported to engage in regular home practice using guided meditation recordings accompanying the treatment manual.

**Session 1 “Awareness and Automatic Pilot”:** The introductory session emphasised that the primary aim of the programme is to cultivate a different way of relating to experience, including difficult thoughts and emotions. It was highlighted that such shifts in perspective may be particularly beneficial during periods of low mood, while also acknowledging that applying these skills may feel more challenging under such conditions. The importance of regular practice was emphasised alongside a compassionate stance towards oneself when intentions are difficult to implement. The structure, content, practices (body scan), and exercises (e.g., raisin exercise) remained unchanged.

**Session 2 “Living in Our Heads”:** Structure and core components (body scan, sitting meditation, and thoughts and feelings exercise) followed the standard protocol. Homework instructions for the pleasant events diary were adapted to encourage awareness of subtle fluctuations in mood, helping participants to notice small positive shifts and reduce the risk of demoralisation in the context of persistent negative affect.

**Session 3 “Gathering the Scattered Mind”:** Teachers were encouraged to use the alternative session structure described in the manual, which places greater emphasis on mindful movement and begins with an extended movement practice. However, they retained discretion to adapt this depending on their clinical judgement and the needs of the group.

**Session 4 “Recognizing Aversion”:** The exercise focusing on recognising negative automatic thoughts as the “voice of depression” was delivered as outlined in the manual. Given the potential difficulty in identifying cognitive biases during acute depressive episodes, teachers were encouraged to support this process through guided dialogue. Exercises were anchored in personally relevant past situations characterised by depressed mood and contrasted with periods of relative wellness. Changes in mood elicited during the reading of automatic thoughts were used to illustrate the impact of negative thinking patterns, followed by guidance in responding with the breathing space. This component was intentionally used as a form of exposure, with careful pacing to minimise distraction. Core practices (hearing meditation, sitting meditation, mindful walking) and other session elements remained unchanged.

**Session 5 “Allowing/Letting Be”:** The structure and content of the session, including “sitting with difficulty” meditation, were retained. Teachers were asked to remain attentive to possible activation of traumatic memories and were provided with additional guidance on managing intrusive imagery where necessary.

**Session 6 “Thoughts are not Facts”:** Standard session content was maintained. The relapse signature exercise was introduced in a way that acknowledged ongoing symptom fluctuation, ensuring relevance for participants experiencing persistent depressive symptoms.

**Session 7 “How Can I Best Take Care of Myself”:** The session followed the standard format. Teachers placed additional emphasis on the role of behavioural activation, highlighting the potential value of activity scheduling within current depressive episodes.

**Session 8 “Maintaining and Extending New Learning”:** The final session was delivered in line with the standard MBCT protocol without modification.

All group sessions were delivered via videoconferencing using a secure online platform (Zoom). Delivery was informed by guidance on maintaining key aspects of mindfulness-based teaching competence in online formats, as outlined in the addendum to the Mindfulness-Based Interventions: Teaching Assessment Criteria (MBI:TAC) (Sansom et al., 2020). While videoconferencing offers advantages in terms of accessibility and scalability, it may also introduce barriers related to access to, and familiarity with, digital technology, particularly for older or economically disadvantaged participants.

**References**

Barnhofer, T., Dunn, B. D., Strauss, C., Ruths, F. A., Barrett, B., Ryan, M., … Warren, F. C. (2025). Mindfulness-based cognitive therapy versus treatment as usual after non-remission with NHS Talking Therapies high-intensity psychological therapy for depression: a UK-based clinical effectiveness and cost-effectiveness randomised, controlled, superiority trial. *The Lancet Psychiatry*, *12*(6), 433–446. <https://doi.org/10.1016/s2215-0366(25)00105-1>

Sansom, S., Crane, R., Karunavira, Koerbel, L., & Yiangou, A. (2020). *Addendum to the Mindfulness-Based Interventions: Teaching Assessment Criteria (MBI:TAC) for assessing online delivery of mindfulness-based programs*. <https://mbitac.bangor.ac.uk/documents/addendum-online-delivery.pdf>

#

# **Supplementary Table 1**

Sociodemographic and clinical characteristics of participants by group

|  | MBCT+TAU  (n = 118) | TAU alone  (n = 116) |
| --- | --- | --- |
| Age, years, M (SD) | 42.9 (13.3) | 42.0 (14.4) |
| Gender |  |  |
| Female | 82 (69.4) | 84 (72.4) |
| Male | 34 (28.8) | 31 (26.7) |
| Other | 0 (0.0) | 1 (0.8) |
| Prefer not to say | 2 (1.6) | 0 (0.0) |
| Marital status |  |  |
| Single | 37 (31.3) | 49 (42.2) |
| Married, civil partnership, cohabiting | 47 (39.8) | 33 (28.4) |
| Separated, divorced, widowed | 16 (13.5) | 18 (15.5) |
| In a long-term relationship | 16 (13.5) | 14 (12.0) |
| Prefer not to say | 2 (1.6) | 2 (1.7) |
| Ethnicity |  |  |
| Asian/Asian British | 2 (1.6) | 5 (4.3) |
| Black/African/Caribbean/Black British | 3 (2.5) | 3 (2.5) |
| Mixed/Multiple ethnic groups | 7 (5.9) | 5 (4.3) |
| Other | 1 (0.8) | 4 (3.4) |
| White | 103 (87.2) | 98 (84.4) |
| Prefer not to say | 2 (1.6) | 1 (0.8) |
| Highest educational attainment |  |  |
| None | 2 (1.6) | 3 (2.5) |
| GCSE* or equivalent | 19 (16.1) | 15 (12.9) |
| A-Level** or equivalent | 29 (24.5) | 26 (22.4) |
| Undergraduate or equivalent | 34 (28.8) | 33 (28.4) |
| Postgraduate or equivalent | 33 (27.9) | 36 (31.0) |
| Prefer not to say | 1 (0.8) | 3 (2.5) |
| Annual household income |  |  |
| £0 to £10,000 | 19 (16.1) | 7 (6.0) |
| £10 001 to £20 000 | 16 (13.5) | 22 (18.9) |
| £20 001 to £30 000 | 14 (11.8) | 24 (20.6) |
| £30 001 to £40 000 | 15 (12.7) | 12 (10.3) |
| £40 001 to £50 000 | 12 (10.1) | 8 (6.8) |
| £50 001 to £60 000 | 12 (10.1) | 8 (6.8) |
| £60 001 to £70 000 | 5 (4.2) | 2 (1.7) |
| £70 001 to £80 000 | 3 (2.5) | 3 (2.5) |
| £80 001 to £90 000 | 4 (3.3) | 1 (0.8) |
| £90 001 to £100 000 | 0 (0.0) | 2 (1.7) |
| £100 001 to £150 000 | 1 (0.8) | 5 (4.3) |
| £150 001 to £200 000 | 1 (0.8) | 0 (0.0) |
| £200 001+ | 0 (0.0) | 0 (0.0) |
| Prefer not to say | 16 (13.5) | 22 (18.9) |
| Previous episodes of depression, M (SD) | 5.43 (10.13) | 6.72 (16.68) |
| Age of onset, M (SD) | 20.69 (10.57) | 19.92 (10.40) |
| Currently taking antidepressant medication | 69 (58.4) | 70 (60.3) |
| Depressive symptoms severity (PHQ-9), M (SD) | 17.95 (3.92) | 17.77 (3.83) |
| PHQ-9 thresholds |  |  |
| Moderate depression | 26 (22.0) | 27 (23.2) |
| Moderately severe depression | 45 (38.1) | 47 (40.5) |
| Severe depression | 47 (39.8) | 42 (36.2) |

*Note*: Unless otherwise stated, reported are respective numbers with percentages in brackets.

PHQ-9 thresholds are 10 to 14 for moderate depression, 15 to 19 for moderately severe depression, and 20 to 27 for severe depression.

# **Supplementary Table 2**

Parameter estimates, standard errors, 95% confidence intervals and significance levels for moderation, mediation, and moderated mediation (a- and b-path) analyses with group effect adjusted for additional trial minimisation variables (trial site and antidepressant use at entry)

| Label | Estimate | SE | 95% CI | *p* |
| --- | --- | --- | --- | --- |
| Moderation |  |  |  |  |
| b1 (Group → T2 PHQ-9) | −2.48 | 0.69 | [−3.84–−1.13] | <.001 |
| b2 (T0 PHQ-9 → T2 PHQ-9) | 0.65 | 0.13 | [0.37–0.88] | <.001 |
| b3 (Group × T0 PHQ-9 → T2 PHQ-9) | −0.08 | 0.18 | [−0.44–0.28] | .661 |
| c1 (Antidepressants → T0 PHQ-9) | −0.20 | 0.72 | [−1.58–1.19] | .773 |
| c2 (Site Devon vs London → T0 PHQ-9) | −0.13 | 0.89 | [−1.89–1.59] | .883 |
| c3 (Site Devon vs Sussex → T0 PHQ-9) | −0.33 | 0.85 | [−2.04–1.37] | .695 |
| Mediation |  |  |  |  |
| a (Group → T1 EQ Decentering) | 3.45 | 0.61 | [2.24–4.65] | <.001 |
| m0 (T0 EQ Decentering → T1 EQ Decentering) | 0.57 | 0.06 | [0.43–0.70] | <.001 |
| m1 (T0 EQ Decentering → Antidepressants) | −0.61 | 0.63 | [−1.95–0.64] | .331 |
| m2 (T0 EQ Decentering → Site Devon vs London) | −0.68 | 0.77 | [−2.19–0.82] | .377 |
| m3 (T0 EQ Decentering → Site Devon vs Sussex) | −0.90 | 0.74 | [−2.36–0.54] | .223 |
| c’ (Group → T2 PHQ-9) | −1.93 | 0.74 | [−3.44–−0.54] | .009 |
| b (T1 EQ Decentering → T2 PHQ-9) | −0.15 | 0.07 | [−0.28–−0.01] | .026 |
| y0 (T0 PHQ-9 → T2 PHQ-9) | 0.59 | 0.09 | [0.41–0.76] | <.001 |
| y1 (T0 PHQ-9 → Antidepressants) | −0.39 | 0.72 | [−1.80–0.97] | .579 |
| y2 (T0 PHQ-9 → Site Devon vs London) | −0.23 | 0.88 | [−1.98–1.47] | .790 |
| y3 (T0 PHQ-9 → Site Devon vs Sussex) | −0.49 | 0.84 | [−2.19–1.18] | .556 |
| a × b (Group → T1 EQ Decentering → T2 PHQ-9) | −0.54 | 0.26 | [−1.06–−0.04] | .039 |
| Moderated Mediation (a-path) * |  |  |  |  |
| a1 (Group → T1 EQ Decentering) | 0.59 | 0.10 | [0.38–0.81] | <.001 |
| a2 (T0 PHQ-9 → T1 EQ Decentering) | 0.06 | 0.05 | [−0.05–0.17] | .294 |
| a3 (Group × T0 PHQ-9 → T1 EQ Decentering) | −0.01 | 0.11 | [−0.22–0.22] | .962 |
| m0 (T0 EQ Decentering → T1 EQ Decentering) | 0.54 | 0.06 | [0.41–0.67] | <.001 |
| ma1 (Antidepressants → T1 EQ Decentering) | −0.11 | 0.11 | [−0.34–0.11] | .327 |
| ma2 (Site Devon vs London → T1 EQ Decentering) | −0.11 | 0.13 | [−0.38–0.14] | .398 |
| ma3 (Site Devon vs Sussex → T1 EQ Decentering) | −0.14 | 0.13 | [−0.40–0.11] | .279 |
| c’ (Group → T2 PHQ-9) | −1.93 | 0.74 | [−3.44–−0.54] | .009 |
| b1 (T1 EQ Decentering → T2 PHQ-9) | −0.89 | 0.40 | [−1.64–−0.08] | .026 |
| y0 (T0 PHQ-9 → T2 PHQ-9) | 2.30 | 0.35 | [1.59–2.96] | <.001 |
| yo1 (Antidepressants → T2 PHQ-9) | −0.40 | 0.72 | [−1.81–0.97] | .578 |
| yo2 (Site Devon vs London → T2 PHQ-9) | −0.23 | 0.88 | [−1.98–1.47] | .789 |
| yo3 (Site Devon vs Sussex → T2 PHQ-9) | −0.49 | 0.84 | [−2.19–1.18] | .556 |
| a × b (Group → T1 EQ Decentering → T2 PHQ-9) | −0.53 | 0.26 | [−1.06–−0.04] | .040 |
| Moderated Mediation (b-path) * |  |  |  |  |
| a (Group → T1 EQ Decentering) | 0.60 | 0.10 | [0.39–0.81] | <.001 |
| m0 (T0 EQ Decentering → T1 EQ Decentering) | 0.53 | 0.06 | [0.40–0.65] | <.001 |
| ma1 (Antidepressants → T1 EQ Decentering) | −0.10 | 0.11 | [−0.34–0.11] | .331 |
| ma2 (Site Devon vs London → T1 EQ Decentering) | −0.11 | 0.13 | [−0.38–0.14] | .378 |
| ma3 (Site Devon vs Sussex → T1 EQ Decentering) | −0.15 | 0.13 | [−0.41–0.09] | .223 |
| c’ (Group → T2 PHQ-9) | −1.93 | 0.72 | [−3.40–−0.56] | .008 |
| b1 (T1 EQ Decentering → T2 PHQ-9) | −0.87 | 0.39 | [−1.62–−0.05] | .027 |
| b2 (T0 PHQ-9 → T2 PHQ-9) | 2.27 | 0.34 | [1.56–2.92] | <.001 |
| b3 (T0 PHQ-9 × T1 EQ Decentering → T2 PHQ-9) | −0.74 | 0.31 | [−1.36–−0.15] | .017 |
| yo1 (Antidepressants → T2 PHQ-9) | −0.41 | 0.71 | [−1.81–0.95] | .565 |
| yo2 (Site Devon vs London → T2 PHQ-9) | −0.14 | 0.87 | [−1.88–1.52] | .872 |
| yo3 (Site Devon vs Sussex → T2 PHQ-9) | −0.48 | 0.83 | [−2.14–1.17] | .560 |
| a × b (Group → T1 EQ Decentering → T2 PHQ-9) | −0.53 | 0.26 | [−1.03–−0.02] | .041 |

*Note*: PHQ-9, Patient Health Questionnaire 9, EQ Decentering, Experiences Questionnaire Decentering subscale.

* Coefficients in the moderated mediation models are reported on a standardised scale, which was used to facilitate estimation of interaction terms and conditional indirect effects; corresponding unstandardised estimates are reported in the primary mediation model.

# **Supplementary Table 3**

Parameter estimates, standard errors, 95% confidence intervals and significance levels for moderation, mediation, and moderated mediation (a- and b-path) analyses with cross-baseline adjustment (both mediator and outcome model are adjusted for both baseline symptoms and baseline decentering) and group effect adjusted for additional trial minimisation variables (trial site and antidepressant use at entry)

| Label | Estimate | SE | 95% CI | p |
| --- | --- | --- | --- | --- |
| Moderation |  |  |  |  |
| b1 (Group → T2 PHQ-9) | −2.48 | 0.69 | [−3.85–−1.14] | <.001 |
| b2 (T0 PHQ-9 → T2 PHQ-9) | 0.66 | 0.13 | [0.39–0.91] | <.001 |
| b3 (Group × T0 PHQ-9 → T2 PHQ-9) | −0.07 | 0.18 | [−0.44–0.29] | .682 |
| d (T0 EQ Decentering → T2 PHQ-9) | 0.06 | 0.07 | [−0.07–0.21] | .390 |
| c1 (Antidepressants →T0 PHQ-9) | −0.18 | 0.72 | [−1.59–1.23] | .799 |
| c2 (Site Devon vs London → T0 PHQ-9) | −0.05 | 0.90 | [−1.84–1.69] | .954 |
| c3 (Site Devon vs Sussex →T0 PHQ-9) | −0.34 | 0.86 | [−2.05–1.38] | .690 |
| Mediation |  |  |  |  |
| a (Group → T1 EQ Decentering) | 3.41 | 0.61 | [2.21–4.58] | <.001 |
| m0 (T0 EQ Decentering → T1 EQ Decentering) | 0.58 | 0.06 | [0.45–0.71] | <.001 |
| mP (T0 PHQ-9→ T1 EQ Decentering) | 0.09 | 0.08 | [−0.07–0.26] | .287 |
| m1 (T0 EQ Decentering → Antidepressants) | −0.63 | 0.64 | [−1.95–0.61] | .318 |
| m2 (T0 EQ Decentering → Site Devon vs London) | −0.65 | 0.77 | [−2.15–0.84] | .399 |
| m3 (T0 EQ Decentering → Site Devon vs Sussex) | −0.83 | 0.75 | [−2.32–0.65] | .271 |
| c’ (Group → T2 PHQ-9) | −1.51 | 0.74 | [−3.01–−0.06] | .041 |
| b (T1 EQ Decentering → T2 PHQ-9) | −0.27 | 0.07 | [−0.40–−0.11] | <.001 |
| y0 (T0 PHQ-9 → T2 PHQ-9) | 0.65 | 0.09 | [0.47–0.82] | <.001 |
| yM (T0 EQ Decentering → T2 PHQ-9) | 0.22 | 0.07 | [0.07–0.37] | .004 |
| y1 (T0 PHQ-9 → Antidepressants) | −0.42 | 0.71 | [−1.80–0.94] | .547 |
| y2 (T0 PHQ-9 → Site Devon vs London) | −0.06 | 0.87 | [−1.73–1.61] | .943 |
| y3 (T0 PHQ-9 → Site Devon vs Sussex) | −0.62 | 0.84 | [−2.34–1.08] | .457 |
| a × b (Group → T1 EQ Decentering → T2 PHQ-9) | −0.93 | 0.31 | [−1.57–−0.36] | .003 |
| Moderated Mediation (a-path)* |  |  |  |  |
| a1 (Group → T1 EQ Decentering) | 0.59 | 0.10 | [0.38–0.81] | <.001 |
| a2 (T0 PHQ-9 → T1 EQ Decentering) | 0.06 | 0.05 | [−0.05–0.17] | .292 |
| a3 (Group × T0 PHQ-9 → T1 EQ Decentering) | −0.01 | 0.11 | [−0.21–0.22] | .968 |
| m0 (T0 EQ Decentering → T1 EQ Decentering) | 0.54 | 0.06 | [0.42–0.67] | <.001 |
| ma1 (Antidepressants → T1 EQ Decentering) | −0.11 | 0.11 | [−0.34–0.10] | .319 |
| ma2 (Site Devon vs London → T1 EQ Decentering) | −0.11 | 0.13 | [−0.38–0.15] | .402 |
| ma3 (Site Devon vs Sussex → T1 EQ Decentering) | −0.14 | 0.13 | [−0.40–0.11] | .275 |
| c’ (Group → T2 PHQ-9) | −1.51 | 0.74 | [−2.98–−0.06] | .042 |
| b1 (T1 EQ Decentering → T2 PHQ-9) | −1.57 | 0.42 | [−2.32–−0.66] | <.001 |
| y0 (T0 PHQ-9 → T2 PHQ-9) | 2.53 | 0.34 | [1.83–3.18] | <.001 |
| y1 (T0 EQ Decentering → T2 PHQ-9) | 1.21 | 0.41 | [0.37–2.00] | .003 |
| yo1 (Antidepressants → T2 PHQ-9) | −0.42 | 0.70 | [−1.80–0.94] | .546 |
| yo2 (Site Devon vs London → T2 PHQ-9) | −0.06 | 0.87 | [−1.73–1.61] | .943 |
| yo3 (Site Devon vs Sussex → T2 PHQ-9) | −0.62 | 0.84 | [−2.34–1.07] | .457 |
| a × b (Group → T1 EQ Decentering → T2 PHQ-9) | −0.93 | 0.31 | [−1.58–−0.36] | .003 |
| Moderated Mediation (b-path)* |  |  |  |  |
| a (Group → T1 EQ Decentering) | 0.59 | 0.10 | [0.38–0.80] | <.001 |
| m0 (T0 EQ Decentering → T1 EQ Decentering) | 0.54 | 0.06 | [0.42–0.67] | <.001 |
| ma1 (Antidepressants → T1 EQ Decentering) | −0.11 | 0.11 | [−0.34–0.10] | .318 |
| ma2 (Site Devon vs London → T1 EQ Decentering) | −0.11 | 0.13 | [−0.37–0.14] | .399 |
| ma3 (Site Devon vs Sussex → T1 EQ Decentering) | −0.14 | 0.13 | [−0.41–0.11] | .271 |
| c’ (Group → T2 PHQ-9) | −1.49 | 0.72 | [−2.97–−0.13] | .039 |
| b1 (T1 EQ Decentering → T2 PHQ-9) | −1.55 | 0.41 | [−2.28–−0.63] | <.001 |
| b2 (T0 PHQ-9 → T2 PHQ-9) | 2.51 | 0.34 | [1.81–3.16] | <.001 |
| b3 (T0 PHQ-9 × T1 EQ Decentering → T2 PHQ-9) | −0.80 | 0.31 | [−1.41–−0.18] | .010 |
| y1 (T0 EQ Decentering → T2 PHQ-9) | 1.22 | 0.40 | [0.42–2.00] | .003 |
| yo1 (Antidepressants → T2 PHQ-9) | −0.44 | 0.70 | [−1.83–0.90] | .525 |
| yo2 (Site Devon vs London → T2 PHQ-9) | −0.04 | 0.86 | [−1.65–1.69] | .957 |
| yo3 (Site Devon vs Sussex → T2 PHQ-9) | −0.62 | 0.82 | [−2.26–1.05] | .453 |
| a × b (Group → T1 EQ Decentering → T2 PHQ-9) | −0.93 | 0.30 | [−1.53–−0.34] | .002 |

*Note*: PHQ-9, Patient Health Questionnaire 9, EQ Decentering, Experiences Questionnaire Decentering subscale.

* Coefficients in the moderated mediation models are reported on a standardised scale, which was used to facilitate estimation of interaction terms and conditional indirect effects; corresponding unstandardised estimates are reported in the primary mediation model.
